# 

**Published:** 1867-01

**Authors:** 


### ORIGINAL CONTRIBUTIONS.

ART. I. THE MECHANICAL TREATMENT NECESSARY IN INFLAMMATION OF THE KNEE-JOINT; WITH A DESCRIPTION OF A NEW APPARATUS FOR MAKING EXTENSION. By JULIEN S. SHERMAN, M.D., Chicago, Ill., ---- 1
ART. II. A PAPER ON EPIDEMICS. By H. NANCE, M.D., Kewanee, Ill. (Read to the Military Tract Medical Society), ---- 5
ART. III. CASE OF OVARIOTOMY. By D. MASON, M.D., Prairie du Chien, Wis., ---- 18
ART. IV. WOUND OF THE INTESTINE—RECOVERY. Reported by Drs. MASON & WHITNEY, Prairie du Chien, Wis., ---- 21
ART. V. ESSAY ON CHOLERA. By N. WRIGHT, M.D., Chatham, Ill., (Read before the Illinois Medical Society, June 6, 1866,) ---- 23
ART. VI. CASE OF DEATH FROM CHLOROFORM. By C. R. PARKE, M.D., Bloomington, Ill. (Read to the Illinois State Medical Society), ---- 33
ART. VII. A CASE OF ELEPHANTIASIS—SUCCESSFUL RECOVERY. By R. DEXTER, M.D., Chicago, ---- 35

### PROCEEDINGS OF SOCIETIES.

Chicago Medical Society, ---- 36
Military Tract Association, ---- 40
Morgan County Medical Society, ---- 43
Quincy Medical Society, ---- 48

### BOOK NOTICES.

The Science and Practice of Medicine. By William Aitken, M.D., Edin., Prof. of Pathology in the Army Medical School, etc., ---- 52
Clinical Notes on Uterine Surgery, with special Reference to the Sterile condition. By J. Marion Sims, A.B., M.D., late Surgeon to Women's Hospital, New York; etc., etc., ---- 53
A Hand-Book of Ophthalmic Surgery, for the use of Practitioners. By J. Z. Laurence, F.R.C.S., M.B., (Uni. Lon.) Surgeon to the Ophthalmic Hospital, Southwark, etc., etc., ---- 53
An Index of Diseases and their Treatment. By Thos. Hawkes Tanner, M.D., F.L.S., Member of the Royal College of Physicians, etc., ---- 53
Conservative Surgery, as Exhibited in Remedying some of the Mechanical Causes that Operate Injuriously both in Health and Disease; with Illustrations. By Henry G. Davis, M.D., Member of the Amer. Med. Association, etc., ---- 53
A Treatise on the Principles and Practice of Medicine; Designed for the use of Practitioners and Students of Medicine. By Austin Flint, M.D., Professor of the Principles and Practice of Medicine in the Bellevue Hospital Medical College, and in Long Island College Hospital, etc., etc., ---- 53
Practical Therapeutics, considered chiefly with Reference to Articles of the Materia Medica. By Edward John Waring, F.R.C.S., F.L.S., Surgeon in Her Majesty's Indian Army, ---- 54

### EDITORIAL.

Chicago Medical Society, ---- 54
Medical Department of University of Michigan, ---- 61
Clinical Items, ---- 62
The Chair of Surgery in Rush Medical College, ---- 63
Mortality for the Month of December, ---- 64
Dr. Conneau, promoted to a seat at the Luxembourg, ---- 64

